# Evaluation of the feasibility of a midwifery educator continuous professional development (CPD) programme in Kenya and Nigeria: a mixed methods study

**DOI:** 10.1186/s12909-024-05524-w

**Published:** 2024-05-14

**Authors:** Duncan N. Shikuku, Hauwa Mohammed, Lydia Mwanzia, Alice Norah Ladur, Peter Nandikove, Alphonce Uyara, Catherine Waigwe, Lucy Nyaga, Issak Bashir, Eunice Ndirangu, Carol Bedwell, Sarah Bar-Zeev, Charles Ameh

**Affiliations:** 1 Liverpool School of Tropical Medicine (Kenya), P.O. Box 24672-00100, Nairobi, Kenya; 2 Liverpool School of Tropical Medicine (UK), Liverpool, L3 5QA UK; 3 Liverpool School of Tropical Medicine (Nigeria), Utako District, P.O Box 7745, Abuja, Nigeria; 4 Moi University, P.O. Box 4606-30100, Eldoret, Kenya; 5 Masinde Muliro University of Science and Technology, P.O. Box 190-50100, Kakamega, Kenya; 6 Maseno University, P.O. Box 3275-40100, Kisumu, Kenya; 7 Kenya Medical Training College, P.O Box 30195-00100, Nairobi, Kenya; 8 Department of Family Health, Ministry of Health (Kenya), P.O. Box 30016-00100, Nairobi, Kenya; 9 Aga Khan University of East Africa, P.O Box 39340-00623, Nairobi, Kenya; 10 Burnet Institute, 85 Commercial Road Prahran Victoria, Melbourne, Australia; 11 University of Nairobi, P. O. Box 19676-00100, Nairobi, Kenya; 12 Diponegoro University, JI. Prof Sudarto No 13, Temalang, Kec, Tembalang, Kota, Semarang, Jawa Tengah 50275 Indonesia

**Keywords:** Midwifery, Education, Continuous professional development, Feasibility, Kenya, Nigeria

## Abstract

**Background:**

Midwifery education is under-invested in developing countries with limited opportunities for midwifery educators to improve/maintain their core professional competencies. To improve the quality of midwifery education and capacity for educators to update their competencies, a blended midwifery educator-specific continuous professional development (CPD) programme was designed with key stakeholders. This study evaluated the feasibility of this programme in Kenya and Nigeria.

**Methods:**

This was a mixed methods intervention study using a concurrent nested design. 120 randomly selected midwifery educators from 81 pre-service training institutions were recruited. Educators completed four self-directed online learning (SDL) modules and three-day practical training of the blended CPD programme on teaching methods (theory and clinical skills), assessments, effective feedback and digital innovations in teaching and learning. Pre- and post-training knowledge using multiple choice questions in SDL; confidence (on a 0–4 Likert scale) and practical skills in preparing a teaching a plan and microteaching (against a checklist) were measured. Differences in knowledge, confidence and skills were analysed. Participants’ reaction to the programme (relevance and satisfaction assessed on a 0–4 Likert scale, what they liked and challenges) were collected. Key informant interviews with nursing and midwifery councils and institutions’ managers were conducted. Thematic framework analysis was conducted for qualitative data.

**Results:**

116 (96.7%) and 108 (90%) educators completed the SDL and practical components respectively. Mean knowledge scores in SDL modules improved from 52.4% (± 10.4) to 80.4% (± 8.1), preparing teaching plan median scores improved from 63.6% (IQR 45.5) to 81.8% (IQR 27.3), and confidence in applying selected pedagogy skills improved from 2.7 to 3.7, *p* < 0.001. Participants rated the SDL and practical components of the programme high for relevance and satisfaction (median, 4 out of 4 for both). After training, 51.4% and 57.9% of the participants scored 75% or higher in preparing teaching plans and microteaching assessments. Country, training institution type or educator characteristics had no significant associations with overall competence in preparing teaching plans and microteaching (*p* > 0.05). Qualitatively, educators found the programme educative, flexible, convenient, motivating, and interactive for learning. Internet connectivity, computer technology, costs and time constraints were potential challenges to completing the programme.

**Conclusion:**

The programme was feasible and effective in improving the knowledge and skills of educators for effective teaching/learning. For successful roll-out, policy framework for mandatory midwifery educator specific CPD programme is needed.

## Introduction

Quality midwifery education underpins the provision of quality midwifery care and is vital for the health and well-being of women, infants, and families [1]. The recent State of the World’s Midwifery report (SoWMy) (2021) indicates that urgent investments are needed in midwifery, especially quality midwifery education, to improve health outcomes for women and neonates. Despite evidence to support midwifery, midwifery education and training is grossly underfunded in low- and middle-income countries (LMICs) with variation in the quality, content and duration of content between and within countries [2]. Barriers to achieving quality education are: inadequate content, lack of learning and teaching materials, insufficient and poorly trained educators and weak regulation, midwifery educators having no connection with clinical practice or opportunities for updating their knowledge or skills competencies [3, 4].

The WHO, UNFPA, UNICEF and the International Confederation of Midwives’ (ICM) seven-step action plan to strengthen quality midwifery education, and ICM’s four pillars for midwives to achieve their potential emphasize strengthening midwifery faculty to teach students as a key priority [4, 5]. Consequently, ICM recommends that (i) at least 50% of midwifery education curriculum should be practise-based with opportunities for clinical experience, (ii) midwifery faculty should use fair, valid and reliable formative and summative assessment methods to measure student performance and progress in learning and (iii) midwifery programmes have sufficient and up-to-date teaching and learning resources and technical support for virtual/distance learning to meet programme needs [6]. To achieve this, WHO’s Midwifery Educator Core Competencies and ICM’s Global Standards for Midwifery Education provide core competencies that midwifery educators must possess for effective practice [6, 7]. The WHO’s global midwifery educator survey in 2018–2019 reported that fewer than half of the educators (46%) were trained or accredited as educators [5]. Educators are important determinants of quality graduates from midwifery programmes [7]. However, the survey identified that none of the educators felt confident in all of WHO’s midwifery educator core competencies [5]. Further evidence shows that many midwifery educators are more confident with theoretical classroom teaching than clinical teaching despite advances in teaching methods and have low confidence in facilitating online/virtual teaching and learning [4, 8, 9]. To remain competent, design and deliver competency-based curriculum and strengthen midwifery practice, ICM and WHO emphasize that midwifery faculty should engage in ongoing professional development as a midwifery practitioner, teacher/lecturer and leader [6, 10, 11]. However in many settings there is inadequate provision or access to faculty development opportunities [12].

## Continuous professional development (CPD)

Continuous professional development has been defined as the means by which members of the profession maintain, improve and broaden their knowledge, expertise, and competence, and develop the personal and professional qualities required throughout their professional lives [13]. This can be achieved through multiple formal educational pathways based on the ICM Global Standards for Midwifery Education whilst incorporating the ICM Essential Competencies for Basic Midwifery Practice [6, 14]. There are formal CPD activities where there is structured learning that often follows set curricula, usually approved by independent accreditation services or informal CPD that is usually self-directed learning. Participating in accredited CPD programmes is beneficial to the profession. A requirement of regular CPD renewal by a country to maintain licensure ensures an up-to-date, relevant nursing and midwifery workforce [15] and increases the legitimacy of CPD [16]. Structured learning (direct or distant), mandatory training, attending workshops and conferences, accredited college/university courses and trainings, research and peer review activities are opportunities for CPD [17]. Importantly, these CPD programmes are essential for safe, competent and effective practice that is essential to the universal health coverage (UHC) & maternal and newborn health SDGs agenda particularly in developing countries [18, 19].

Whilst regulatory bodies and employers in many countries have requirements for midwives to complete CPD programmes and activities, these programmes and supporting activities are found to be ineffective if CPD is irrelevant to the practitioners’ practice setting, attended only because of monetary or non-monetary benefits, geared towards improving a skill for which there is no demonstrated need, and taken only to meet regulatory requirements rather than to close a competency gap [20]. In most LMICs, midwifery licensure is permanent, without obligation to demonstrate ongoing education or competence [15]. Consequently, CPD processes are not in place, and if in place, not fully utilised. A systematic review on CPD status in WHO regional office for Africa member states reported that nurses and midwives are required to attend formalised programmes delivered face-to-face or online, but only16 out of 46 (34.7%) member states had mandatory CPD programmes [15]. This underscores the need for designing regulator approved midwifery educator CPD programmes to improve the quality of midwifery education in LMICs.

### Modes and approaches for delivery of CPD

Face-to-face contact is a common mode of delivery of CPD although mHealth is an emerging platform that increases access, particularly to nurses and midwives in rural areas [12, 21]. Emerging platforms and organisations such as World Continuing Education Alliance (WCEA) offer mHealth learning opportunities in LMICs for skilled health personnel to access CPD resources that can improve health care provider knowledge and skills and potentially positively impact healthcare outcomes [22]. Although there is evidence of capacity building initiatives and CPD for midwifery educators in LMICs [23], these have been largely delivered as part of long duration (2-year) fellowship programmes and led by international organisations. In addition, these programmes have largely focused on curriculum design, leadership, management, research, project management and programme evaluation skills in health professions education with little on teaching and learning approaches and assessment for educators [24–26]. Successful CPD initiatives should be (i) accredited by the national regulatory bodies (Nursing and Midwifery Councils); (ii) multifaceted and provide different types of formal and informal learning opportunities and support; (iii) combine theory and clinical practice to develop the knowledge, skills and attitudes and (iv) must be adapted to fit the local context in which participants work and teach to ensure local ownership and sustainability of the initiatives [16].

Short competency-based blended trainings for educators improve their competence and confidence in delivering the quality midwifery teaching. However, systems for regular updates to sustain the competencies are lacking [27, 28]. Evidence on effectiveness of the available CPD initiatives is limited. Even where these initiatives have been evaluated, this has largely focused on the outcomes of the programmes and little attention on the feasibility and sustainability of such programmes in low-resourced settings [24, 25, 29]. As part of global investments to improve the quality of midwifery education and training, Liverpool School of Tropical Medicine (LSTM) in collaboration with the UNFPA Headquarters Global Midwifery Programme and Kenya midwifery educators developed a blended midwifery educator CPD programme (described in detail in the methods section). The CPD programme modules in this programme are aligned to the WHO’s midwifery educators’ core competencies [7] and ICM essential competencies for midwifery practice [14]. The programme is also aligned to the nursing and midwifery practice national regulatory requirements of Nursing and Midwifery Councils in LMICs such as Kenya and Nigeria, and relevant national policy [30–32].This programme aimed at sustaining and improving the educators’ competencies in delivery of their teaching, assessments, mentoring and feedback to students. To promote uptake, there is need to test the relevance and practicability of the CPD programme. Feasibility studies are used to determine whether an intervention is appropriate for further testing, relevant and sustainable in answering the question – Can it work [33]? The key focus of these studies are acceptability of the intervention, resources and ability to manage and implement intervention (availability, requirements, sustainability), practicality, adaptation, integration into the system, limited efficacy testing of the intervention in controlled settings and preliminary evaluation of participant responses to the intervention [33–35].

This study evaluated the feasibility of the LSTM/UNFPA midwifery educator CPD programme using the Kirkpatrick’s model for evaluating training programmes [36]. This model is an effective tool with four levels for evaluating training programmes. Level 1 (Participants’ reaction to the programme experience) helps to understand how satisfying, engaging and relevant participants find the experience. Level 2 (Learning) measures the changes in knowledge, skills and confidence after training. Level 3 (Behaviour) measures the degree to which participants apply what they learned during training when they are back on job and this can be immediately and several months after the training. This level is critical as it can also reveal where participants might need help to transfer learning during the training to practice afterwards. Level 4 (Results) measures the degree to which targeted outcomes occur because of training. In this study, participants’ reaction to the programme – satisfaction and relevance of the programme to meeting their needs (level 1) and change in knowledge, confidence and skills after the CPD programme (level 2) were assessed. Also, user perspectives and barriers to implementing the CPD programme were explored.

## Methods

### Study design

This was a mixed methods intervention study using a concurrent nested/embedded/convergent design conducted in Kenya and Nigeria in May and June 2023. This was designed to evaluate the feasibility of the midwifery educator CPD programme. The goal was to obtain different but complementary data to better understand the CPD programme with the data collected from the same participants or similar target populations [37].

The quantitative component of the evaluation used a quasi-experimental pre-post and post-test only designs to evaluate the effectiveness of the blended CPD programme intervention among midwifery educators from mid-level training colleges and universities from the two countries. Pre and post evaluation of knowledge (online self-directed component) and skills (developing a teaching plan during the face-to-face component) was performed. Post intervention evaluation on programme satisfaction, relevance of CPD programme and microteaching sessions for educators was conducted.

The qualitative component of the evaluation included open-ended written responses from the midwifery educators and master trainers to describe what worked well (enablers), challenges/barriers experienced in the blended programme and key recommendations for improvement were collected. In addition, key informant interviews with the key stakeholders (nursing and midwifery councils and the national heads of training institutions) were conducted. Data on challenges anticipated in the scale up of the programme and measures to promote sustainability, access and uptake of the programme were collected from both educators and key stakeholders.

A mixed methods design was used for its strengths in (i) collecting the two types of data (quantitative and qualitative) simultaneously, during a single data collection phase, (ii) provided the study with the advantages of both quantitative and qualitative data and (iii) helped gain perspectives and contextual experiences from the different types of data or from different levels (educators, master trainers, heads of training institutions and nursing and midwifery councils) within the study [38, 39].

### Setting

The study was conducted in Kenya and Nigeria. Kenya has over 121 mid-level training colleges and universities offering nursing and midwifery training while Nigeria has about 300. Due to the vastness in Nigeria, representative government-owned nursing and midwifery training institutions were randomly selected from each of the six geo-political zones in the country and the Federal Capital Territory. Mid-level training colleges offer the integrated nursing and midwifery training at diploma level while universities offer integrated nursing and midwifery training at bachelor/master degree level in the two countries (three universities in Kenya offer midwifery training at bachelor level). All nurse-midwives and midwives trained at both levels are expected to possess ICM competencies to care for the woman and newborn. Midwifery educators in Kenya and Nigeria are required to have at least advanced diploma qualifications although years of clinical experience are not specified.

It is a mandatory requirement of the Nursing and Midwifery Councils for nurse/midwives and midwifery educators in both countries to demonstrate evidence of CPD for renewal of practising license in both countries [40, 41]. A minimum of 20 CPD points (equivalent to 20 credit hours) is recommended annually for Kenya and 60 credit hours for Nigeria every three years. However, there are no specific midwifery educator CPD that incorporated both face-to-face and online modes of delivery, available for Kenya and Nigeria and indeed for many countries in the region. Nursing and midwifery educators are registered and licensed to practice nursing and midwifery while those from other disciplines who teach in the midwifery programme are qualified in the content they teach.

### Study sites

In Kenya, a set of two mid-level colleges (Nairobi and Kakamega Kenya Medical Training Colleges (KMTCs) and two universities (Nairobi and Moi Universities), based on the geographical distribution of the training institutions were identified as CPD Centres of Excellence (COEs)/hubs. In Nigeria, two midwifery schools (Centre of Excellence for Midwifery and Medical Education, College of Nursing and Midwifery, Illorin, Kwara State and Centre of Excellence for Midwifery and Medical Education, School of Nursing Gwagwalada, Abuja, FCT) were identified. These centres were equipped with teaching and EmONC training equipment for the practical components of the CPD programme. The centres were selected based on the availability of spacious training labs/classes specific for skills training and storage of equipment and an emergency obstetrics and newborn care (EmONC) master trainer among the educators in the institution. They were designated as host centres for the capacity strengthening of educators in EmONC and teaching skills.

### Intervention

Nursing and midwifery educators accessed and completed 20 h of free, self-directed online modules on the WCEA portal and face-to-face practical sessions in the CPD centres of excellence.

### The design of the midwifery educator CPD programme

**Fig. 1:**
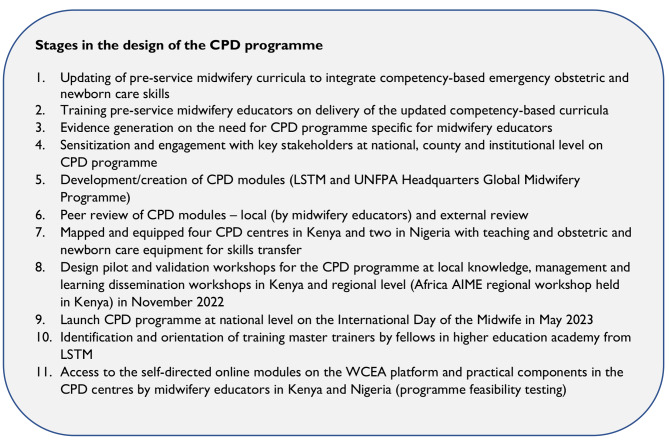
Midwifery educator CPD programme design stages

The design of the CPD modules was informed by the existing gap for professional development for midwifery educators in Kenya and other LMICs and the need for regular updates in knowledge and skills competencies in delivery of teaching [9, 15, 23, 28]. Liverpool School of Tropical Medicine led the overall design of the nursing and midwifery educator CPD programme (see Fig. 1 for summarised steps taken in the design of the blended programme).

This was a two-part blended programme with a 20-hour self-directed online learning component (accessible through the WCEA platform at no cost) and a 3-day face-to-face component designed to cover theoretical and practical skills components respectively. The 20-hour self-directed online component had four 5-hour modules on reflection practice, teaching/learning theories and methods, student assessments and effective feedback and mentoring. These modules had pretest and post-test questions and were interactive with short videos, short quizzes within modules, links for further directed reading and resources to promote active learning. This online component is also available on the WCEA platform as a resource for other nurses and midwifery educators across the globe (https://wcea.education/2022/05/05/midwifery-educator-cpd-programme/ ).

Practical aspects of competency-based teaching pedagogy, clinical teaching skills including selected EmONC skills, giving effective feedback, applying digital innovations in teaching and learning for educators and critical thinking and appraisal were delivered through a 3-day residential face-to-face component in designated CPD centres of excellence. Specific skills included: planning and preparing teaching sessions (lesson plans), teaching practical skills methodologies (lecture, simulation, scenario and role plays), selected EmONC skills, managing teaching and learning sessions, assessing students, providing effective feedback and mentoring and use of online applications such as Mentimeter and Kahoot in formative classroom assessment of learning. Selected EmONC skills delivered were shoulder dystocia, breech delivery, assisted vaginal delivery (vacuum assisted birth), managing hypovolemic shock and pre-eclampsia/eclampsia and newborn resuscitation. These were designed to reinforce the competencies of educators in using contemporary teaching pedagogies. The goal was to combine theory and practical aspects of effective teaching as well as provide high quality, evidence-based learning environment and support for students in midwifery education [4]. These modules integrated the ICM essential competencies for midwifery practice to provide a high quality, evidence-based learning environment for midwifery students. The pre and post tests form part of the CPD programme as a standard assessment of the educators.

As part of the design, this programme was piloted among 60 midwifery educators and regulators from 16 countries across Africa at the UNFPA funded Alliance to Improve Midwifery Education (AIME) Africa regional workshop in Nairobi in November 2022. They accessed and completed the self-directed online modules on the WCEA platform, participated in selected practical sessions, self-evaluated the programme and provided useful feedback for strengthening the modules.

The Nursing and Midwifery Councils of Kenya and Nigeria host the online CPD courses from individual or organisation entities on the WCEA portal. In addition, the Nursing Council of Kenya provides opportunities for self-reporting for various CPD events including accredited online CPD activities/programmes, skill development workshops, attending conferences and seminars, in-service short courses, practice-based research projects (as learner, principal investigator, principal author, or co-author) among others. In Nigeria, a certificate of attendance for Mandatory Continuing Professional Development Programme (MCPDP) is required as evidence for CPD during license renewal. However, the accredited CPD programmes specific for midwifery educators are not available in both countries and Africa region [15, 42].

### Participants and sample size

Bowen and colleagues suggest that many feasibility studies are designed to test an intervention in a limited way and such tests may be conducted in a convenience sample, with intermediate rather than final outcomes, with shorter follow-up periods, or with limited statistical power [34].

A convenience random sample across the two countries was used. Sample size calculations were performed using the formula for estimation of a proportion: a 95% confidence interval for estimation of a proportion can be estimated using the formula: $p\pm 1.96\sqrt{\frac{\text{p}(1-\text{p})}{n}}$ The margin of error (d) is the second term in the equation. For calculation of the percentage change in competence detectable Stata’s power paired proportion function was used.

To achieve the desired level of low margin of error of 5% and a 90% power (value of proportion) to detect competence change after the training, a sample of 120 participants was required. Using the same sample to assess competence before and after training, so that the improvement in percentage competent can be derived and 2.5% are assessed as competent prior to training but not after training (regress), a 90% power would give a 12% improvement change in competence after the training.

**Fig. 2:**
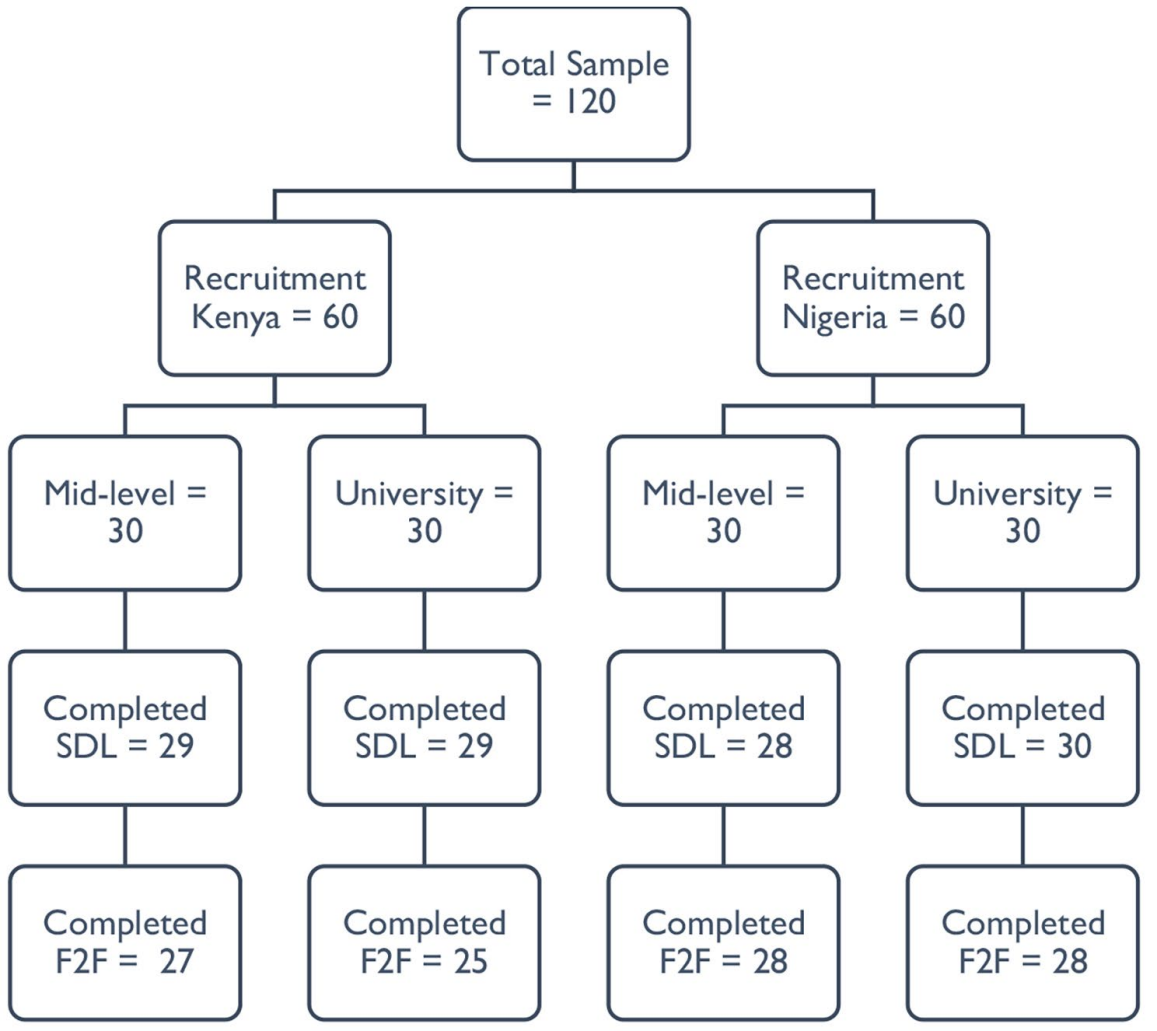
Flow of participants in the CPD programme (SDL = self-directed online learning; F2F = face-to-face practical)

A random sample of 120 educators (60 each from Kenya & Nigeria; 30 each from mid-level training colleges and universities) were invited to participate via an email invitation in the two components of the CPD programme (Fig. 2). Importantly, only participants who completed the self-directed online modules were eligible to progress to the face-to-face practical component.

For qualitative interviews, eight key informant interviews were planned with a representative each from the Nursing and Midwifery Councils, mid-level training institutions’ management, university and midwifery associations in both countries. Interviews obtained data related to challenges anticipated in the scale up of the programme and measures to promote sustainability, access and uptake of the programme.

### Participant recruitment

Only nursing and midwifery educators registered and licensed by the Nursing and Midwifery Councils were eligible and participated. This was because they can access the WCEA website with the self-directed online programme via the Nursing and Midwifery Councils’ websites, only accessible to registered and licensed nurses and midwives.

The recruitment process was facilitated through the central college management headquarters (for mid-level training colleges’ educators) and Nursing and Midwifery Councils (for university participants). Training institutions’ heads of nursing and midwifery departments were requested to share the contact details of all educators teaching midwifery modules, particularly the antepartum, intrapartum, postpartum and newborn care modules in the two countries. A list of 166 midwifery educators from 81 universities and mid-level training colleges was obtained through the Heads of the Department in the institutions.

The research lead, with the assistance by the co-investigator from Nigeria then randomly sampled 120 educators based on institution type and region for representativeness across the countries. Following the selection of participants, the two investigators shared the electronic detailed participant study information sheet and consent form to the potential participants one week before the start of the self-directed online modules. Clear guidance and emphasis on the conduct of the two-part program including completing the mandatory four self-directed online modules was provided. Due to the large number of eligible participants, the recruitment and consenting process was closed after reaching the first 30 participants consenting per institution type and region, with 1–2 educators per institution randomly recruited. This allowed as many institutions to be represented across the country as possible. Participants received a study information sheet and an auto-generated copy of the electronic consent form completed in their emails. Other opportunities for participating in the two-part programme were provided as appropriate for those who missed out. Only those who completed the four online modules were invited for the practical component. A WhatsApp community group for the recruited participants was formed for clarifications about the study, troubleshooting on challenges with online access and completion of the modules before and during the programme.

### Self-directed online component

Upon consenting, the contact details of the educators from each level were shared with WCEA program director for generation of a unique identification code to access the self-directed online modules on the WCEA portal. Educators completed their baseline characteristics (demographic and academic) in the online platform just before the modules. Each self-directed online module was estimated to be completed in five hours. Only after completing a module was the participant allowed to progress to the next module. The modules were available for participants to complete at their own time/schedule. An autogenerated certificate of completion with the participant’s post-completion score was awarded as evidence of completing a module. Participants completed a set of 20 similar pretest and posttest multiple choice questions in each module for knowledge check. A dedicated staff from WCEA actively provided technical support for educators to register, access and complete the online modules. At the end of each module, participants completed a self-evaluation on a 5-point Likert scale for satisfaction (0 = very unsatisfied, 1 = unsatisfied, 2 = neutral, 3 = satisfied and 4 = very satisfied) and relevance of the modules (0 = very irrelevant, 1 = irrelevant, 2 = neutral, 3 = relevant and 4 = very relevant). This provided participants’ reactions to the different components of the modules on whether they met the individual educator’s development needs. In addition, participants responded to the open-ended questions at the end of the modules. These were on what they liked about the modules, challenges encountered in completing the modules and suggestions for improvement of the modules. A maximum period of two weeks was given for educators to complete the modules before progressing to the practical component.

### Practical component

The practical component was delivered by a pool of 18 master trainers who received a 1-day orientation from the research lead before the training. The master trainers were a blend of experienced midwifery and obstetrics faculty in teaching and clinical practice actively engaged in facilitating EmONC trainings selected from Kenya and Nigeria. Four of these master trainers from Kenya participated in the delivery of both sets of trainings in Kenya and Nigeria.

Only educator participants who completed the self-directed online modules and certified were invited to participate in a 3-day residential practical component. Two separate classes were trained (mid-level and university level educators) per country by the same group of eight master trainers. The sessions were delivered through short interactive lectures; small group and plenary discussions; skills demonstrations/simulations and scenario teaching in small breakout groups; role plays and debrief sessions. Sessions on digital innovations in teaching and learning were live practical sessions with every participant using own laptop. Nursing and Midwifery Councils representatives and training institutions’ managers were invited to participate in both components of the programme.

Participant costs for participating in the two-part CPD programme were fully sponsored by the study. These were internet data for completing the self-directed online component and residential costs – transport, accommodation, and meals during the practical component.

### Data collection

Self-directed online knowledge pretests and post-tests results, self-rated measures of satisfaction and relevance of the modules including what they liked about the modules, challenges encountered in accessing and completing the modules and suggestions for improvement data was extracted from the WCEA platform in Microsoft Excel.

On day 1 of the practical component, participants using their personal computers developed a teaching plan. On the last day (day 3), participants prepared a teaching plan and powerpoint presentation for the microteaching sessions. No teaching plan template from the trainers was provided to the participants before the training. However, they used formats from their institutions if available. A standard teaching plan template was provided at the end of the training.

The group of master trainers and participants were divided into groups for the microteaching sessions which formed part of the formative assessment. Each participant delivered a powerpoint presentation on a topic of interest (covered in the teaching plan) to the small group of 13–15 participants. This was followed by a structured session of constructive feedback that started with a self-reflection and assessment. This was followed by peer supportive and constructive feedback from the audience participants and faculty/master trainers identifying areas of effective practice and opportunities for further development. Each microteaching session lasted 10–15 min. Each of the microteaching session presentation and teaching plan were evaluated against a pre-determined electronic checklist by two designated faculty members independently during/immediately after the microteaching session. The checklist was adapted from LSTM’s microteaching assessment of the United Kingdom’s Higher Education Academy (HEA)’s Leading in Global Health Teaching (LIGHT) programme. The evaluation included preparing a teaching plan, managing a teaching and learning session using multiple interactive activities, designing and conducting formative assessments for learning using digital/online platforms, and giving effective feedback and critical appraisal. The master trainers received an orientation training on the scoring checklist by the lead researcher/corresponding author.

Self-rated confidence in different teaching pedagogy skills were evaluated before (on day 1) and after (day 3) the training on a 5-point Likert scale (0 = not at all confident, 1 = slightly confident, 2 = somewhat confident, 3 = quite confident and 4 = very confident). A satisfaction and relevance of practical component evaluation on a 5-point Likert scale was completed by the participants on an online designed form on day 3 after the microteaching sessions of the practical component. This form also had a similar qualitative survey with open-ended questions on what they liked about the practical component, challenges encountered in completing the practical component and suggestions for improvement of the component.

Using a semi-structured interview guide, six qualitative key informant interviews, each lasting about 30–45 min, were conducted by the lead researcher with the Nursing and Midwifery Councils focal persons and training institutions’ managers. These were audio recorded in English, anonymized, and deleted after transcription. These interviews were aimed at getting their perspectives on the programme design, anticipated barriers/enablers with the CPD programme and strategies for promoting uptake of the CPD programme. These interviews were considered adequate due to their information power (indicating that the more information the sample holds, relevant for the actual study, the lower amount of participants is needed) [43] and upon obtaining data saturation, considered the cornerstone of rigor in qualitative research [44, 45].

### Assessment of outcomes

Participants’ reaction to the programme (satisfaction and relevance) (Kirkpatrick level 1) was tested using the self-rated 5-point Likert scales. Change in knowledge, confidence and skills (Kirkpatrick level 2) was tested as follows: knowledge through 20 pretest and post-test multiple choice questions per module in the self-directed online modules; confidence in applying different pedagogy skills through the self-rated 5-point Likert scale; and teaching skills through the observed microteaching sessions using a checklist.

### Reliability and validity of the data collection tools

The internal consistency (a measure of the reliability, generalizability or reproducibility of a test) of the Likert scales/tools assessing the relevance of the online and practical modules and satisfaction of educators with the two blended modules were tested using the Cronbach’s alpha statistic. The Cronbach’s alpha statistics for the four Likert scales/tools ranged from 0.835 to 0.928, all indicating acceptably good to excellent level of reliability [46]. Validity (which refers to the accuracy of a measure) of the Likert scales were tested using the Pearson correlation coefficient statistic. Obtained correlation values were compared to the critical values and p-values reported at 95% confidence intervals. All the scales were valid with obtained Pearson correlation coefficients reported − 0.1946, which were all greater than the critical values (*p* < 0.001) [46]. The semi-structured interview guides for the qualitative interviews with the training institutions’ managers and midwifery councils (regulators) were developed and reviewed by expert study team members with experience in qualitative research.

### Data management and analysis

Data from the online/electronic tools was extracted in Microsoft Excel and exported to SPSS version 28 for cleaning and analysis. Normality of data was tested using the Kolmogorov-Smirnov test suitable for samples above 50. Proportions of educator characteristics in the two countries were calculated. Differences between the educator characteristics in the two countries were tested using chi-square tests (and Fishers-exact test for cells with counts of less than 5).

For self-rated relevance of CPD programme components and satisfaction with the programme on the 0–4 Likert scales, descriptive statistics were calculated (median scores and proportions). Results are presented as bar graphs and tables. Cronbach alpha and Pearson correlation coefficients were used to test the reliability and validity of the test items respectively.

Change in knowledge in online modules, confidence in pedagogy skills and preparing teaching plans among educators was assessed by comparing pre-training scores and post-training scores. Descriptive statistics are reported based on normality of data. Differences in the scores were analysed using the Wilcoxon signed ranks tests, a non-parametric equivalent of the paired t-test. Differences between educators scores in microteaching by country and institution type were performed by Mann-Whitney U test. Level of competence demonstrated in the teaching plan and microteaching skill was defined as the percentage of the desired characteristics present in the teaching plan and microteaching session, set at 75% and above. The proportion of participants that achieved the desired level of competence in their teaching plan and microteaching skill was calculated. Binary logistic regression models were used to assess for the strengths of associations between individual educator and institutional characteristics (age, gender, qualifications, length of time as educator, training institution and country) and the overall dichotomised competent score (proportion achieved competence in teaching plan and microteaching skills). P-values less than 0.05 at 95% confidence interval were considered statistically significant.

Preparation for qualitative data analysis involved a rigorous process of transcription of recorded interviews with key informants. In addition, online free text responses by midwifery educators on what worked well, challenges encountered, and recommendations were extracted in Microsoft Excel format and exported to Microsoft Word for data reduction (coding) and theme development. Qualitative data was analysed using thematic framework analysis by Braun and Clarke (2006) as it provides clear steps to follow, is flexible and uses a very structured process and enables transparency and team working [47]. Due to the small number of transcripts, computer assisted coding in Microsoft Word using the margin and comments tool were used. The six steps by Braun and Clarke in thematic analysis were conducted: (i) familiarising oneself with the data through transcription and reading transcripts, looking for recurring issues/inconsistencies and, identifying possible categories and sub-categories of data; (ii) generating initial codes – both deductive (using topic guides/research questions) and inductive coding (recurrent views, phrases, patterns from the data) was conducted for transparency; (iii) searching for themes by collating initial codes into potential sub-themes/themes; (iv) reviewing themes by generating a thematic map (code book) of the analysis; (v) defining and naming themes (ongoing analysis to refine the specifics of each sub-theme/theme, and the overall story the analysis tells); and (vi) writing findings/producing a report. Confidentiality was maintained by using pseudonyms for participant identification in the study. Trustworthiness was achieved by (i) respondent validation/check during the interviews for accurate data interpretation; (ii) using a criterion for thematic analysis; (iii) returning to the data repeatedly to check for accuracy in interpretation; (iv) quality checks and discussions with the study team with expertise in mixed methods research [39, 47].

Integration of findings used the parallel-databases variant and are synthesised in the discussion section. In this common approach, two parallel strands of data are collected and analysed independently and are only brought together during interpretation. The two sets of independent results are then synthesized or compared during the discussion [39].

## Results

### Quantitative findings

#### Midwifery educators’ characteristics

A total of 116 (96.7%) and 108 (90.0%) educators from 81 institutions completed the self-directed online learning and practical component respectively from the two countries. There were no significant differences between countries in educators’ qualifications, when last taught a midwifery class and whether attended any CPD training in the preceding year before the study (*p* > 0.05). Overall, only 28.7% of the educators had a midwifery related CPD training in the preceding year before the study. Midwifery educator characteristics are outlined below (Table 1).

**Table 1:** Midwifery educators’ characteristics

Characteristic		Kenya (*N* = 58)	Nigeria (*N* = 58)	Total (*N* = 116)	*P*-value
Face-to-face component					
Age (years)	31–40	15 (25.9%)	12 (20.7%)	27 (23.3%)	
	41–50	28 (48.3%)	20 (34.5%)	48 (41.4%)	
	51–60	8 (13.8%)	13 (22.4%)	21 (18.1%)	
	Over 61	5 (8.6%)	4 (6.9%)	9 (7.8%)	
	Missing	2 (3.4%)	9 (15.5%)	11 (9.5%)	0.115
Gender	Male	16 (27.6%)	6 (10.3%)	22 (19.0%)	
	Female	42 (72.4%)	52 (89.7%)	94 (81.0%)	0.018*
Training institution	Mid-level	29 (50.0%)	28 (48.3%)	57 (49.1%)	
	University	29 (50.0%)	30 (51.7%)	59 (50.9%)	0.853
Qualifications	Diploma	3 (5.2%)	2 (3.4%)	5 (4.3%)	
	Degree	15 (25.9%)	18 (31.0%)	33 (28.4%)	
	Masters	33 (56.9%)	30 (51.7%)	63 (54.3%)	
	PhD	7 (12.1%)	8 (13.8%)	15 (12.9%)	0.877
Years as midwifery educator	Less than 1	3 (5.2%)	2 (3.4%)	5 (4.3%)	
	1–5 years	14 (24.1%)	13 (22.4%)	27 (23.3%)	
	6–10 years	26 (44.8%)	13 (22.4%)	39 (33.6%)	
	11–15 years	9 (15.5%)	11 (19.0%)	20 (17.2%)	
	Over 15 years	4 (6.9%)	10 (17.2%)	14 (12.1%)	
	Missing	2 (3.4%)	9 (15.5%)	11 (9.5%)	0.038*
When last taught a class (months)	Less than 1	40 (69.0%)	41 (70.7%)	81 (69.8%)	
	1–3 months	6 (10.3%)	2 (3.4%)	8 (6.9%)	
	4–6 months	2 (3.4%)	2 (3.4%)	4 (3.4%)	
	Over 6 months	0 (0.0%)	1 (1.7%)	1 (0.9%)	
	Missing	10 (17.2%)	12 (20.7%)	22 (19.0%)	0.526
Face-to-face practical component		**Kenya (** *N* ** = 52)**	**Nigeria (** *N* ** = 56)**	**Total (** *N* ** = 108)**	
Previous CPD in last 1yr	Yes	11 (21.2%)	20 (35.7%)	31 (28.7%)	
	No	41 (78.8%)	36 (64.3%)	77 (71.3%)	0.095
Institutional teaching plan template available	Yes	28 (53.8%)	12 (21.4%)	40 (37.0%)	
	No	24 (46.2%)	44 (78.6%)	68 (63.0%)	< 0.001*

*P < 0.05 statistically significant

#### Self-directed online component

##### Change in knowledge

This was assessed in each of the four self-directed online modules. The results from ranked scores based on Wilcoxon signed ranks test showed significant improvements in educators’ knowledge in all the four online modules completed (*p* < 0.001). The highest mean score improvement was observed in students’ assessment module, 48.1% (SD ± 15.1) to 85.2% (SD ± 15.7), a 37.1% improvement. Improvements in knowledge in the other modules were as follows: reflective practice (27.6%), mentoring and giving effective feedback (27.4%) and teaching methods (19.2%). Overall knowledge score for all modules improved from 52.4% (SD ± 10.4) to 80.4 (SD ± 8.1), *p* < 0.001 (Table 2).

**Table 2:** Pretest and post-test scores in the self-directed online modules

	Pretest		Post-test			
	Mean	SD	Mean	SD	Z^b^	*P*-value
Reflective practice	51.1	15.9	78.7	9.2	-8.846	< 0.001*
Teaching methods	57.2	13.1	76.4	12.0	-8.532	< 0.001*
Students’ assessments	48.1	15.1	85.2	15.7	-8.561	< 0.001*
Mentoring and giving effective feedback	52.0	14.6	79.4	11.5	-9.027	< 0.001*
Average knowledge score	**52.4**	**10.4**	**80.4**	**8.1**	**-8.895**	< 0.001*

Z^b^ scores based on negative ranks; *P < 0.05 statistically significant

#### Relevance of self-directed online modules

The internal consistency of each of the four modules was tested with Cronbach’s alpha. The overall Cronbach’s alpha for the four items was 0.837, a good and acceptable level of reliability. All the four modules assessed were valid with calculated Pearson correlation coefficient values greater than the critical value of 0.1946 (*p* < 0.001) at 95% confidence interval.

**Fig. 3:**
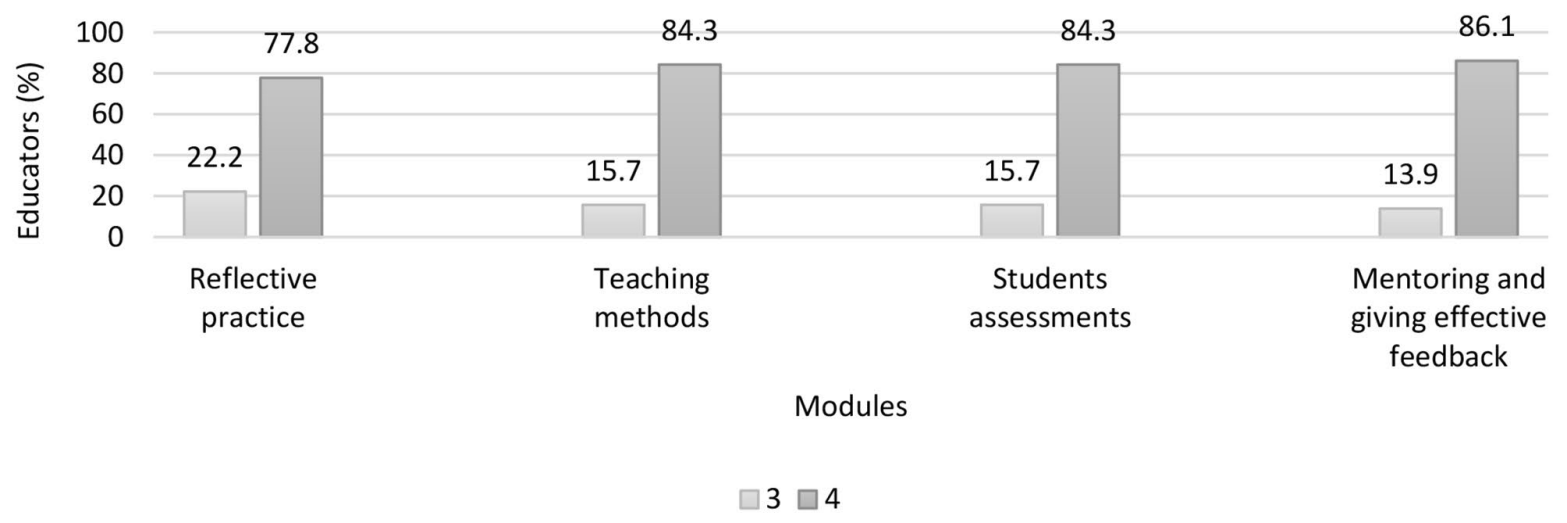
Educators’ ratings of the relevance of self-directed online modules

Educators from the two countries, on a scale of 0–4 rated the online modules as very relevant with a median score of 4 out of 4 (IQR 0) for each of the four modules: reflective practice, teaching methods, students’ assessments and mentoring and giving effective feedback. There were no ratings of 0, 1 and 2 for all the modules (Fig. 3).

#### Satisfaction with the self-directed online modules

The internal consistency of each of the eight items was tested with Cronbach’s alpha. The overall Cronbach’s alpha for the eight items was 0.928, an excellent level of reliability. All the eight items assessed were valid with their obtained Pearson correlation coefficient values greater than the critical value of 0.1946 (*p* < 0.001) at 95% confidence interval.

Each of the eight items rated on satisfaction had a median score of 4 out of 4 (IQR 0). Over 80% of the educators were very satisfied with the online modules’ content as presented in a logical format and informative. Also, the modules helped them to learn something new, updated their knowledge and the materials were useful and valuable for their practice. Over 70% were very satisfied with the modules as they helped them refresh their knowledge and skills with the links and activities embedded in the modules useful in adding to their learning. None of the educators were dissatisfied (rated 0 or 1) with the online modules (Table 3).

**Table 3:** Educators’ satisfaction with the self-directed online modules

Satisfaction statement	Ratings (0–4)			Median (IQR)
	**2**	**3**	**4**	
The module introduction, aims and learning outcomes reflected the module content	2 (1.9%)	36 (33.3%)	70 (64.8%)	4 (0)
The module content was informative	2 (1.9%)	17 (15.7%)	89 (82.4%)	4 (0)
The module content was presented in a logical format	3 (2.8%)	18 (16.7%)	87 (80.6%)	4 [1]
The links and activities added to the learning	3 (2.8%)	29 (26.9%)	76 (70.4%)	4 (0)
I undertook this module to refresh my knowledge and skills	2 (1.9%)	20 (18.5%)	86 (79.6%)	4 (0)
I undertook this module to learn something new	3 (2.8%)	17 (15.7%)	88 (81.5%)	4 (0)
The module met my needs for identifying new knowledge and skills or updating knowledge and skills	2 (1.9%)	18 (16.7%)	88 (81.5%)	4 (0)
The educational materials were useful and valuable	2 (1.9%)	15 (13.9%)	91 (84.3%)	4 (0)

#### Practical component

##### Change in confidence in different pedagogy skills

The internal consistency of each of the eight items assessed was tested with Cronbach’s alpha using the baseline data. The overall Cronbach’s alpha for the eight items was 0.893, a good level of reliability. All the eight items assessed were valid with their obtained Pearson correlation coefficient values greater than the critical value of 0.1946 (*p* < 0.001) at 95% confidence interval.

Changes in confidence before and after the training were compared using the Wilcoxon signed rank test, a parametric equivalent of the paired t-test when data is not normally distributed. The mean score of self-rated confidence of educators on a scale of 0–4 for all the eight skills significantly improved after the training from 2.73 (SD ± 0.68) to 3.74 (SD ± 0.34) (*p* < 0.001). Mean confidence was highest in facilitating a lecture (3.23, SD ± 0.8) and lowest on using digital innovations (Mentimeter) in formative assessment of teaching/learning (1.75, SD ± 1.15) before the training. These improved significantly after the training to 3.84 (SD ± 0.41) for facilitating a lecture and 3.50 (SD ± 0.63) for using digital innovations (Mentimeter) in formative assessment of teaching/learning, *p* < 0.001. The mean confidence of educators was largely average before the training and significantly improved after the training in six skills (*p* < 0.001). These were designing learning outcomes using measurable Bloom’s taxonomy verbs, preparing a teaching plan, identifying relevant resources to enhance learning, facilitating a scenario teaching, facilitating a practical simulation/demonstration and giving effective feedback for learning (Table 4).

**Table 4:** Midwifery educators’ confidence in pedagogy skills before and after training

	Before		After		Z value	*P*-value
Skill	Mean (0–4)	SD	Mean (0–4)	SD		
Preparing a teaching plan	2.75	0.89	3.80	0.43	-7.930	< 0.001*
Designing learning outcomes using Bloom’s taxonomy verbs	2.50	0.89	3.71	0.48	-7.945	< 0.001*
Identifying relevant resources/aids to enhance learning sessions	2.95	0.83	3.78	0.46	-7.092	< 0.001*
Facilitating a lecture	3.23	0.80	3.84	0.41	-6.505	< 0.001*
Facilitating a scenario session	2.88	0.84	3.71	0.53	-6.940	< 0.001*
Simulation/demonstration of a practical skill	2.94	0.86	3.69	0.52	-6.856	< 0.001*
Using digital innovations (Mentimeter) in formative assessment of teaching/learning	1.75	1.15	3.50	0.63	-8.425	< 0.001*
Giving effective feedback for learning	2.81	0.91	3.88	0.38	-7.757	< 0.001*
Overall confidence for 8 items	2.73	0.68	3.74	0.34	-8.789	< 0.001*

*P < 0.05 statistically significant

#### Preparing a teaching plan and microteaching skills

The overall median score in preparing a teaching plan was 63.6% (IQR 45.5) before the training and improved significantly to 81.8% (IQR 27.3) after the training, *p* < 0.001. The median scores differed significantly by country before and after the training. Before the training, Kenyan educators had higher median scores (72.7%, IQR 27.3) compared to Nigeria counterparts (54.5%, IQR 36.4), *p* < 0.001. After the training, Kenyan educators had significantly higher median scores (81.2%, IQR 18.2) than Nigerian counterparts (72.7%, IQR 18.2), *p* = 0.024. However, there were no significant differences in the median scores between the training institutions before and after the training, *p* > 0.05. For microteaching, the overall median score was 76.5% (IQR 29.4). There were no significant differences between countries and training institutions in the microteaching scores, *p* > 0.05. Kenya educators (82.4%, IQR 29.4) had slightly higher scores than Nigeria (76.5%, IQR 29.4), *p* = 0.78. Mid-level educators (79.4%, IQR 29.4) had slightly higher scores than university educators (76.5%, IQR 28.7), *p* = 0.515 (Table 5).

The inter-rater reliability/agreement of the eight pairs of assessors in both countries were assessed by Cohen Kappa statistic. The Kappa statistics for the eight pairs ranged between 0.806 and 0.917, *p* < 0.001, showing near perfect agreement between the pairs of assessors.

**Table 5:** Preparing teaching plan and microteaching skills scores before and after training by country and training institution

	Before			After		
	Median	IQR	*P*-value	Median	IQR	*P*-value
Preparing a teaching plan						
Kenya	72.7	27.3		81.8	18.2	
Nigeria	54.5	36.4	**< 0.001**	72.7	18.2	**0.024**
Mid-level colleges	63.6	34.1		72.7	18.2	
University	63.6	45.5	0.925	81.8	27.3	0.378
Overall*	63.6	45.5	-	81.8	27.3	**< 0.001**
Microteaching						
Kenya				82.4	29.4	
Nigeria				76.5	29.4	0.780
Mid-level colleges				79.4	29.4	
University				76.5	28.7	0.515
Overall*				76.5	29.4	

*P* < 0.05 statistically significant; *Wilcoxon signed rank test performed

#### Association between independent educator and institutional characteristics and the microteaching skill scores

Categorised skills scores (≥ 75% mean score as competent) showed that 55 (51.4%) and 62 (57.9%) of the educators scored 75% or higher in the teaching plan preparation and microteaching skill assessments respectively. Logistic regression analysis showed that educator’s country, age, gender, qualifications, training institution type and length as educator were not significantly associated with the overall categorised teaching plan or microteaching scores (*p* > 0.05).

##### Relevance of the practical component

The internal consistency of each of the six skills items was tested with Cronbach’s alpha. The overall Cronbach’s alpha for the six items was 0.866, a good level of reliability. All the six skills items assessed were valid with their obtained Pearson correlation coefficient values greater than the critical value of 0.1946 (*p* < 0.001) at 95% confidence interval.

**Fig. 4:**
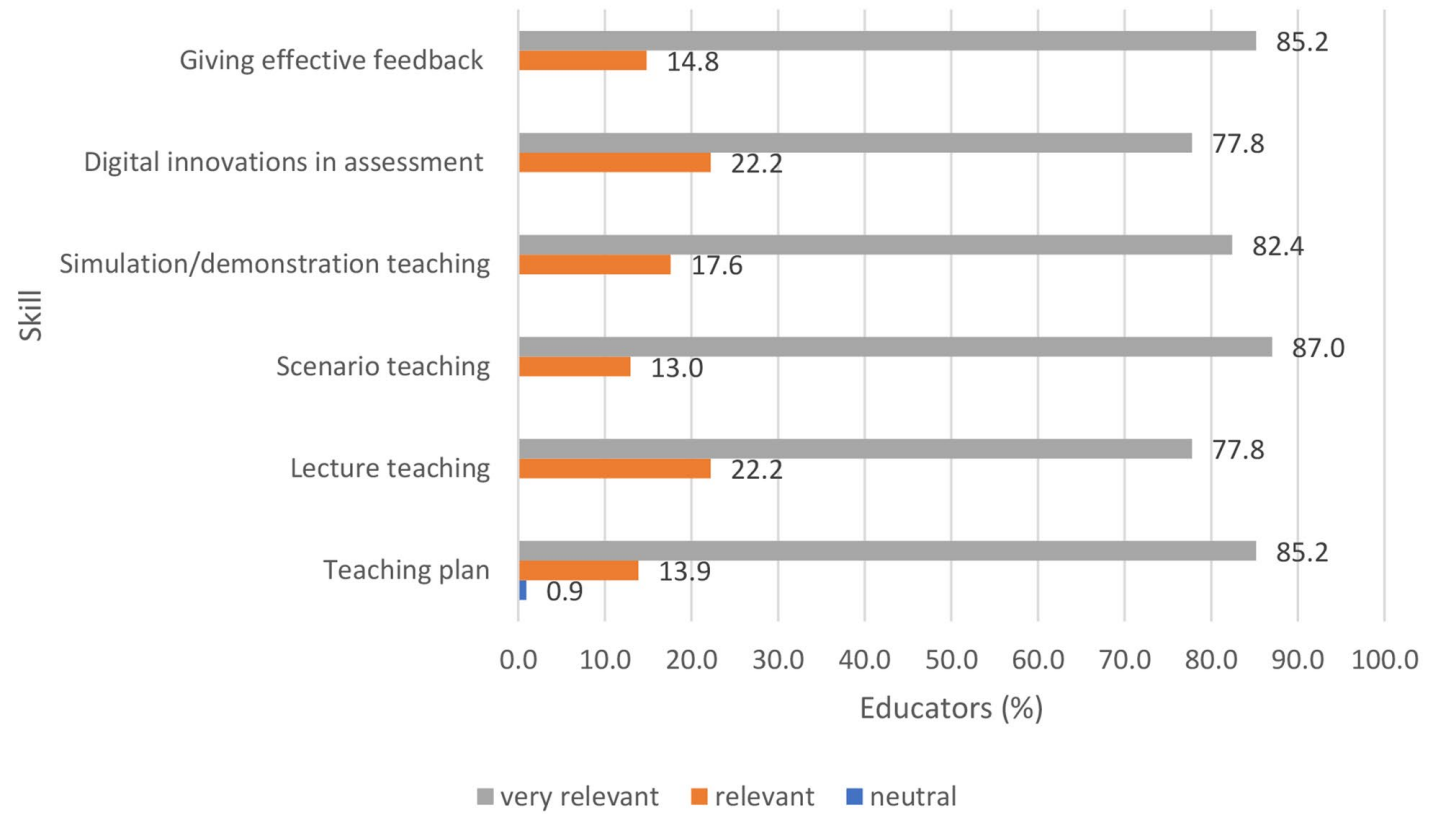
Relevance of the practical components

On a self-rating Likert scale of 0–4, the median score for each of the six skills assessed and trained was 4 out of a maximum of 4, indicating that the educators found the different pedagogy skills very relevant after the training. Over 80% of the educators rated the sessions on teaching plan (85.2%), scenario teaching (87.0%), simulation/demonstration teaching (82.4%) and giving effective feedback (85.2%) as very relevant. Over three-quarters (77.8%) of the educators rated the sessions on lecture teaching and use of digital innovations (Mentimeter) in assessment as very relevant (Fig. 4).

#### Satisfaction with the practical component

The internal consistency of each of the six skills items was tested with Cronbach’s alpha. The overall Cronbach’s alpha for the six items was 0.835, a good level of reliability. All the six skills items assessed were valid with their obtained Pearson correlation coefficient values greater than the critical value of 0.1946 (*p* < 0.001) at 95% confidence interval.

**Fig. 5:**
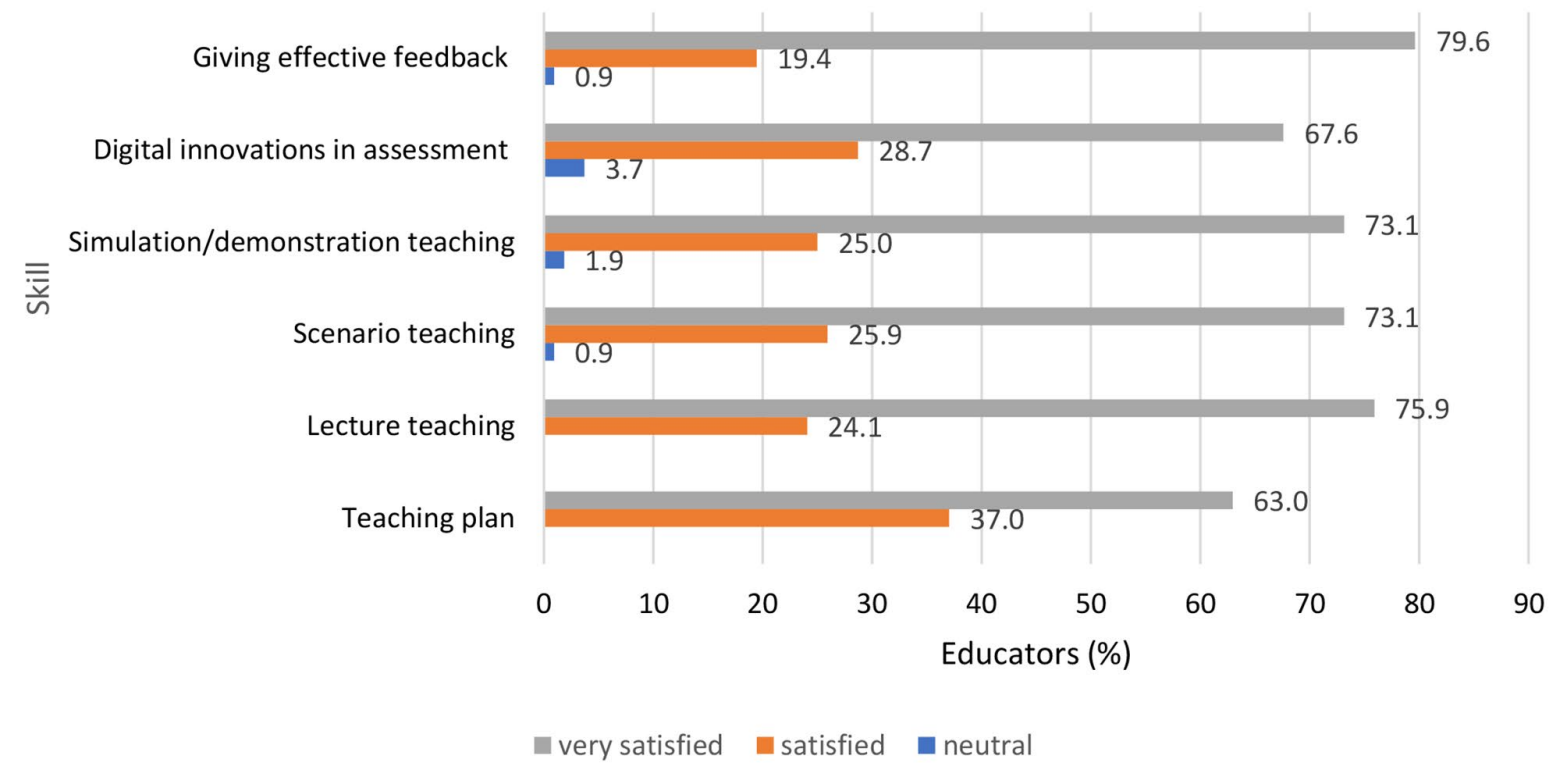
Satisfaction with practical skills

On a self-rating Likert scale of 0–4, the median score for each of the six skills assessed was 4 out of a maximum of 4, indicating that educators were very satisfied with the practical skills sessions. Over 70% of the educators were very satisfied with the sessions on giving effective feedback (79.6%), lecture teaching (75.9%), scenario and simulation teaching (73.1% each). Two-thirds of the educators (67.6%) were very satisfied with the digital innovations in teaching (use of Mentimeter) for formative assessment in teaching and learning. All educators were satisfied with the preparing of teaching plan in teaching and learning with the majority (63.0%) as very satisfied while the remaining 37.0% satisfied. None of the educators were dissatisfied with the practical component of the training (Fig. 5).

## Qualitative findings

### What educators liked about the self-directed online modules

Educators from both levels and countries had similar views on the online component. These are broadly summarised under the sub-themes: (i) educative and relevant for practice, (ii) flexible and convenient learning and (iii) motivating, interesting and interactive.

#### Educative and relevant for practice

Educators reported the online modules as educative and informative and, improved their knowledge in teaching, assessments, reflective practice and providing effective feedback to students to promote learning as well as increasing their self-confidence and critical thinking skills. Besides, educators found the modules valuable and relevant for their professional growth and practice.

*“The modules were well organized, they were relevant to my practice and met my expectations”* university midwifery educator, Kenya.

*“The materials are very rich with current information to guide. Very informative & valuable to my professional growth”* university midwifery educator, Nigeria.

#### Flexible and convenient learning

Educators reported that they could access and complete the online modules at their flexible and convenient time. This flexibility enhanced and stimulated them to complete the informative modules at their comfort times either at home or office without disruption to their schedules.

*“(The modules) gave me ample time to read at my own pace and time without any hurry to understand the content well. They were well organised. Also, flexibility of learning and the access to materials was excellent”* university midwifery educator, Kenya.

*“It is flexible and convenient. It empowers the learner to take ownership of the learning process. Learning is personalized”* mid-level training college midwifery educator, Nigeria.

#### Motivating, interesting and interactive

Educators reported that the online modules were well structured, motivating, interesting and had components that promoted interaction for learning. For example, pretests, various quizzes within the modules and posttest questions and the added specific short extra reading segments promoted interaction and learning.

*“The intermittent assessment questions. It helped maintain my focus”* university *midwifery educator, Nigeria*.

*“Very interactive. They were very informative and extra reading assignments complemented the content” university midwifery educator, Kenya*.

### Challenges encountered with the self-directed online learning modules

Four sub-themes emerged that summarised the challenges experienced by midwifery educators in the two countries to access and complete the self-directed online modules. These are (i) network/internet connectivity, (ii) technology challenges, (iii) electricity power supply and power outages and, (iv) time constraints.

#### Network/internet connectivity

Network and internet connectivity difficulties and fluctuations was the commonest reported challenge in completing the self-directed online modules by educators from both countries. This affected the access, progress, downloading extra resources embedded within the modules and completing the integrated evaluations within the modules.

*“Accessing the modules, problem with submitting forms and exams, had network problem” mid-level training college midwifery educator, Nigeria*.

*“I kept going offline and I would have to restart every time. They were too internet dependent”* university midwifery educator, Kenya.

#### Technology challenges

Technological challenges were observed as well as reported among educators from both countries. These ranged from poor access to emails due to forgotten email addresses, usernames or passwords, difficult access and navigation through the online modules, completing the matching questions that required dragging items, completing the evaluations and downloading certificates after completion of the modules.

*“I am not very good with ICT, so I had issues using my laptop”* mid-level training college midwifery educator, Nigeria.

*“Accessibility was difficult. I had to restart the process a number of times. The modules would sometimes take you back more than 20 slides which delayed the completion rate”* university midwifery educator, Kenya.

#### Electricity power supply interruptions and fluctuations

Power interruptions, fluctuations and outages especially in Nigeria were cited as a challenge to complete the online modules. This delayed the completion of the modules as electric power was critical to access and complete the modules on either WCEA app on mobile phones or computers.

*“The modules should not start from beginning whenever there is interrupted power supply”* MLC midwifery educator, Nigeria.

*“Network failure due to interrupted power supply”* university midwifery educator, Nigeria.

#### Time constraints

Although educators commented the flexibility with which to complete the online modules, time to complete the online modules was also cited as a challenge in both countries.

*“It requires a lot of time, this is a challenge because I am also involved with other activities at the place of work which require my attention”* university midwifery educator, Kenya.

### What educators liked about the practical component

Educators written feedback on what they liked about the practical component of the CPD programme was categorised into the four sub-themes: new knowledge and relevant for practice; improved knowledge, skills and confidence to teach; enhanced participatory and active learning; individualised support in learning.

#### New knowledge and relevant for practice

The practical component provided new learning particularly on the use of digital platforms (Mentimeter and Kahoot) for formative assessment to evaluate learning during classroom teaching. In their integrated teaching using both online and face-to-face delivery, use of technology (Mentimeter and Kahoot) in classroom assessment was not a common practice as most of them had not heard about the available online platforms. They found Mentimeter (and Kahoot) to be interesting resources for formative assessments in class to facilitate teaching and learning. The techniques of giving effective feedback using the sandwich and ‘stop, start, continue’ methods were viewed to promote interaction between the educator and the learner for effective learning. Educators also acknowledged new knowledge and skills updates on EmONC relevant for their practice.

*“Giving feedback, innovation of the online formative assessment, the teaching plan. I wish we would adapt them for daily application rather than the traditional teacher centered one.” Mid-level training college educator, Kenya*.

*“(I liked) Everything, especially the technological innovations for assessment” Mid-level training college educator, Nigeria*.

#### Improved knowledge, skills and confidence to teach

Educators reported that the practical sessions were interactive and engaging with good combination of theory and practice which facilitated learning. They reported that participating in the practical component enabled them to update and improve their knowledge, skills and confidence in planning and delivering theoretical and practical teaching using multiple methods. Similar improvements were reported on preparing and conducting students’ assessments and giving effective feedback to promote learning. On use of technology in formative assessments, the interactive practical sessions boosted the confidence of educators in using Mentimeter (and Kahoot) online platforms during classroom teaching.

*“It helped build my confidence, had hands on practice on clinical skills and teaching skills, learnt about outdated practices and current evidence based clinical and teaching skills.” Mid-level training college educator, Nigeria*.

*“They were very interesting especially the scenarios and skills. I was able to enhance my practical skills and technology in evaluating learning.” University midwifery educator, Kenya*.

#### Enhanced participatory and active learning

The practical component complemented the self-directed online learning for educators. They highly commented and benefitted from the hands-on opportunities to actively engage through return demonstrations during the practical programme. This component also enabled them to brainstorm and contribute actively during the sessions. They highlighted that the practical component enhanced and reinforced learning through active participation in demonstrations, questions, group discussions and plenary sessions.

*“This face-to-face module provided me with the opportunity to brainstorm with other educators, facilitators and resource persons. This will enhance my teaching skills.” Mid-level training college midwifery educator, Nigeria*.

*“Interaction with facilitators who could clarify points that I had earlier not understood, interaction with other participants and was also able to learn from them.” University midwifery educator, Kenya*.

#### Individualised support in learning

Educators received individualised peer support and learning during the practical component. They had opportunities within the small breakout groups for peer learning and one-to-one support from the facilitators to update and learn new knowledge and skills.

*“A chance to get immediate feedback was availed by the presenters.” University midwifery educator, Kenya*.

*“Facilitators were well informed and gave learners opportunity for return demonstration and support.” Mid-level training college midwifery educator, Kenya*.

### Challenges encountered with the practical component

Key challenges reported by the mixed group of educators and master trainers across the two countries include: inadequate time, computer technology challenges and poor internet connectivity for practical components.

#### Inadequate time

Although small breakout sessions were utilised to provide each educator with an opportunity to practice the skills, it was commonly reported that time was inadequate for skills demonstrations and return demonstrations by all educators. This was especially for areas educators had inadequate knowledge and new skills that were observed thus adequate time for teaching and repeat demonstrations for mastery was required. Similar observations were made by the master trainers who felt that some educators had never encountered some of the basic EmONC skills demonstrated or never practised and thus required a longer duration for familiarisation and practice.

“Time was short hence not enough to return demo” *Mid-level training college midwifery educator, Kenya*.

*“Some of the things were new and required more time for demonstration and practice.” Mid-level training college midwifery educator, Nigeria*.

#### Computer technology challenges and poor internet connectivity for practical components

Some educators encountered technical difficulties in using computers during the practical component. In some cases, this was compounded by poor network/internet connectivity. This delayed completion of practical components requiring the use of computers including pretests, preparing teaching plans and presentations, post-tests and classroom demonstrations using digital innovations in teaching and learning. However, assistance was provided by the trainers as appropriate to those who needed technical support.

*“(There were) technical challenges with use of computers for few participants.” Master trainer, Nigeria*.

*“Slow internet can hinder smooth flow of sessions.” Master trainer, Kenya*.

### Key areas for additional support

For quality education and training, master trainers generally recommended that all educators should be trained and regularly supported in the basic EmONC course to strengthen their competencies for effective teaching of EmONC skills. Further support in computer technology use including basics in navigation around windows/programmes, formatting in Microsoft Office Word and Powerpoint, literature searching, and referencing were other critical components to be strengthened.

### Perspectives from training institutions managers and midwifery regulators

#### Measures to ensure midwifery educators take specific CPDs that have been designed to improve their teaching competencies

Key informant interviews with the pre-service training institutions’ managers and nursing and midwifery councils from the two countries were conducted and revealed key strategies outlined below that should ensure access and completion of the blended CPD programme specific for educators’ teaching competencies.

#### Awareness creation, integrating programme into policy and performance appraisal

The aspect of online CPD was highlighted as a new concept in Nigeria. Due to this novelty, the country was reluctant to accredit many online CPD programmes for in-service and pre-service nursing and midwifery personnel. However, the regulatory Nursing and Midwifery Council of Nigeria had established monitoring mechanisms to evaluate its uptake to meet the definition of CPD and is still work in progress.

*“For the online, it’s actually a relatively new concept, in fact because of monitoring and evaluation, we have struggled with accepting online CPDs… So, we’re struggling on how to develop a guideline for online CPDs. So, we’re now starting with the WCEA. So far, only the WCEA has that approval to provide CPD…We said let’s look at how this works out before we can extend it to other providers.” Nursing and Midwifery Council, Nigeria*.

Both countries emphasized the need to create awareness of the CPD programme for midwifery educators and a policy framework for CPD. Regulators emphasized the need to have the CPD programme as mandatory for all midwifery educators through a policy directive. They suggested that the blended CPD programme should form a mandatory specified proportion of the content addressing their specific competencies. Besides, the training institution recommended that the programme should form part of the educator’s performance appraisal on a regular basis. Active monitoring systems were suggested to be in place to ensure compliance of participation and completion to acquire specific relevant competencies in pedagogy.

*“…Ensure that educators take the particular modules before license renewal. Tie modules that are related to midwifery education to the educators and make them mandatory. Yes, we make it as a matter of policy that you should be taking these courses over and over again.” Nursing and Midwifery Council, Nigeria*.

#### Incentives

It was strongly suggested that attaching incentives as motivators to completing the programme would attract educators to complete the CPD programme. These incentives include certification, recognition for participation in curriculum reviews, national examination setting, facilitating national examinations, promotion and service and eligibility as trainers of trainers to colleagues.

*“You attach a course, one training, you cannot guarantee that these courses will be taken. So we find a way to attach something to it. You must have evidence that you attended these programs. So once you attach something like that, they will all flock because there is an incentive to it. Because we say, as an educator, before you go after every examination to examine students, you must have taken these courses.” Nursing and Midwifery Council, Nigeria*.

#### Internet connectivity

Training institutions’ managers suggested investments in internet connectivity for training institutions to support educators access and complete the self-directed online programme. This was also highlighted as a critical challenge for the online component by the educators in both countries.

*“The issues of internet connectivity and I think we need to be proactive about it so that we have a way to constantly bring it to the forefront especially in our policies. But connectivity would be a major area to look at as people are using their money.” Mid-level training college manager, Kenya*.

#### Anticipated challenges in the scale-up of the CPD programme

Key challenges anticipated in the roll-out and scale-up of the blended CPD programme were identified as inadequate skills of the educators in the use of information and communication technology during the practical component (including preparation of powerpoint presentations and completing tasks using a computer), and participant costs to attend the practical component (including participants’ residential costs and investments in proctor technology for ensuring academic integrity and monitoring and evaluation tool for educators’ compliance.) It was also emphasized that due to low remuneration of the educators, additional costs from their pocket to undertake the CPD could be a limiting factor for the intended faculty development initiatives. Other challenges included maintaining quality and academic integrity of the programme, potential bias in the selection of educators to attend future CPD programmes that is based on pre-existing relationships and ensuring an adequate pool of in-country trainers of trainers with midwifery competencies to deliver the practical component of the CPD programme.

There were strong suggestions that personal commitment by educators was required for personal and professional development. There were observations that educators sometimes completed the professional development programmes purely for relicensing and not necessarily for professional development. Regulators and institutional managers emphasized that educators need to understand the value of continuous professional development and create time to participate in the targeted CPD programmes to improve their competencies.

*“We do advise our nurses, or we continue to inform them that taking these courses shouldn’t be tied to license renewal. It shouldn’t be tied to licence expiration or renewal of licences. You should continue to take these courses to develop yourself and not waiting until your licence expired before you take the courses. Yes, we actually try as much as possible to dissociate the renewal of licences with these courses.” Nursing and Midwifery Council, Nigeria*.

## Discussion

### Key results

Our study evaluated the feasibility of what the authors believe to be the first blended programme with online and face-to-face learning available in Africa, as a tool to reach midwifery educators in both urban and rural low-resource areas. In addition, our study is in line to an important call by WHO, UNFPA, UNICEF and ICM for an effective midwifery educator with formal preparation for teaching and engages in ongoing development as a midwifery practitioner, teacher/lecturer and leader [6, 7]. Consequently, our intervention is part of investments for improving and strengthening the capacity of midwifery educators for quality and competent midwifery workforce as recommended by multiple global reports [4, 5, 11] and other publications [12, 15, 23, 42]. Our study findings showed that the midwifery educators were very satisfied with the blended CPD programme. Educators rated the programme as highly relevant, educative, flexible, interesting and interactive, improved their knowledge, confidence and practical skills in their professional competencies for practice. Use of digital technology in teaching and students’ assessment was found to be an effective and innovative approach in facilitating teaching and learning. Key challenges experienced by educators included deficiencies in computer technology use, internet/network connectivity for online components, time constraints to complete the blended programme and isolated electric power outages and fluctuations which affected completion of the self-directed online components. Costs for participating and completing the programme, motivation, investments in information and communication technology, quality assurance and academic integrity were highlighted as critical components for the scale-up of the programme by institutional managers and training regulators. Establishment of a policy framework for educators to complete mandatory specific and relevant CPD was recommended for a successful roll-out in the countries.

### Interpretation of our findings

Our study findings demonstrated that educators found the theoretical and practical content educative, informative and relevant to their practice. Recent evidence showed that midwifery educators had no/limited connection with clinical practice or opportunities for updating their knowledge or skills [15, 42]. This underscores the value and importance of regular opportunities of CPD specific for educators to improve their professional competencies. It has provided these educators with a flexible educational model that allows them to continue working while developing their professional practice.

The use of a blended programme was beneficial as educators’ needs were met. It provided opportunities for educators to reflect, critically think, internalise and complement what was learned in the self-directed online component during the practical phase. This approach has been considered a means to adequately prepare midwifery faculty and improving national midwifery programmes in low-resource and remote settings [48, 49]. Use of self-directed online platforms has emerged as a key strategy to improve access to CPD with flexibility and convenience as educators take responsibility for their own learning. Evidence suggests that the flexibility of net-based learning offers the midwifery educators a new and effective educational opportunity that they previously did not have [50, 51]. A practical – based learning is important in pre-service education settings where the capacity of midwifery educators needs to be strengthened [52, 53]. However, without continuous regular training, the midwives’ competence deteriorate and this in turn threaten the quality of pre-service midwifery education [52, 54]. Implementation of this flexible blended educational model allows educators to continue working while developing their professional practice.

The quality of educators is an important factor affecting the quality of graduates from midwifery programmes to provide quality maternal and newborn health services [7]. Evidence suggests that many midwifery educators are more confident with theoretical classroom teaching than clinical practice teaching and that they also struggle to maintain their own midwifery clinical skills [4, 5]. Our findings showed that the programme was effective, and educators improved their knowledge, confidence and skills in teaching, students’ assessment, effective feedback, reflective practice, mentoring and use of digital innovations in teaching and assessments. Our findings are similar to other related models of capacity building midwifery educators in other developing countries [24, 50, 53, 55–57]. It is expected that educators will apply the learning in their planning for teaching, delivery of interactive and stimulatory teaching, monitoring learning through formative and summative assessments and mentoring their students into competent midwives. This is a pathway for accelerating the achievement of maternal and newborn health SDGs, universal health coverage, ending preventable maternal mortalities and every newborn action plan targets.

The value for CPD on educators’ knowledge, confidence and skills has been demonstrated with opportunities for improvement. Specific CPD targeted to relevant professional competencies is beneficial to the profession, quality of graduates for maternal and newborn health care and global targets. However, further investments in strengthening capacity of educators in EmONC skills and information and communication technology for effective teaching and learning is mandatory. Related challenges with individual technical capacity, technological deficiencies and infrastructure to support the technological advancement have been reported in other studies that have used a blended learning approach [58]. Resource constraints – financial and infrastructural (e.g. computers) as well as internet access are key challenges to participation in CPD activities especially the self-directed learning [16]. Designing self-directed modules that can be accessed and completed offline will increase access especially in poorly connected settings with electric power and network coverage.

### Strengths and limitations

This study assessed the feasibility a blended midwifery educator CPD programme in low resource settings. This was conducted in a multi-country and multi-site context which provided opportunities for learning across the two countries, two levels of training institutions and specific in-country experiences [20]. The study served to improve awareness of the availability of the CPD programme so that (1) regulators can ensure that midwifery educators take this as part of mandatory CPD required for relicensing and (2) training institutions can plan to support their educators access/participate in the practical components of the programme after the study. It is a mandatory requirement of the Nursing and Midwifery Councils of Kenya and Nigeria for nurse/midwives and midwifery educators to demonstrate evidence of CPD for renewal of practising license [40, 41]. The use of mixed methods research design with multiple evaluations was relevant to address the aims and objectives of the study and ensure methodological rigour, depth and scientific validity as recommended for good practice in designing pilot studies [37, 38]. This also enhanced triangulation of findings and enabled the capturing of broad perspectives important in strengthening sustainable implementation of the blended CPD programme [39]. Preliminary findings were disseminated to participant stakeholders from Kenya and Nigeria at the knowledge management and learning event in Nairobi. This approach enhanced the credibility and trustworthiness of the final findings reported. We believe our study findings from different participants using multiple data collection methods are robust, transparent and trustworthy for generalization to other contexts [38].The self-directed learning component of the blended CPD programme is hosted on the WCEA platform which is accessible to healthcare professionals in over 60 countries in Africa, Asia and Middle East and accredited for continuous professional development (59). Although our sample size was small, it is sufficient, geographically representative for training institutions across the countries and acceptable for feasibility studies [34].

The additional cost analysis of implementing the blended midwifery educator CPD programme is relevant and key to the uptake, scale-up and sustainability of the programme but this was not conducted due to limited funding. Different CPD programme funding models exist. In Nigeria, educators are required to meet the costs for accessing and completing the CPD programme components, while in Kenya the cost of accessing the online component is minimal (internet access costs only) and the face-to-face component has to be funded. The cost of implementing the programme should be explored in future studies and optional models for sustainable funding explored with stakeholders.

### Implications

Our findings show demand for the CPD programme. Regular continuous professional development could help to bridge the gap between theory and practice and improve the quality of teaching by midwifery educators. A blended CPD programme is effective in improving the teaching and clinical skills of midwifery educators and increasing their confidence in effective teaching. However, midwifery educators require motivation and close support (individual capacity, time, technological infrastructure and policy) if the blended CPD approach is to be mandatory and successfully implemented in resource limited settings. Besides, regular quality assurance modalities including review of content, monitoring and evaluation of uptake of the CPD programme should be undertaken to ensure that updated and relevant content is available.

For quality CPD programmes, hands-on teaching is more effective than didactic classroom teaching and should be used when feasible to transfer clinical skills. Distance education models (self-directed learning) in combination with short residential training and mentoring should be embraced to strengthen capacity strengthening of midwifery educators; and CPD programmes must consider the local context in which participants work and teach [16, 23]. Evidence has shown that knowledge and clinical skills are retained for up to 12 months after training [54]. Taking the CPD programme annually will potentially maintain/improve knowledge, skills and practice by midwifery educators for quality teaching and learning leading to a competent midwifery workforce.

For quality midwifery education and practice, educators need contact with clinical practice to strengthen classroom teaching [6, 7]. This will promote and enable students to acquire the skills, knowledge, and behaviours essential to become autonomous midwifery practitioners. Therefore, demonstrating relevant practical clinical CPD should be included in midwifery educator CPD policy. In addition, a business case by the CPD hubs on the sustainability of the face-to-face practical components in the centres is necessary. Stakeholder engagement on cost and sustainability are required as key policy components for the scale-up of the blended midwifery educator CPD programme for impact.

## Conclusion

The blended CPD programme was relevant, acceptable and feasible to implement. Midwifery educators reacted positively to its content as they were very satisfied with the modules meeting their needs and rated the content as relevant to their practice. The programme also improved their knowledge, confidence and skills in teaching, students’ assessments and providing effective feedback for learning and using digital/technological innovations for effective teaching and learning. Investments in information and communication technology, quality assurance and academic integrity were highlighted as critical components for the scale-up of the programme. For successful and mandatory implementation of the specific midwifery educator CPD programme to enhance practice, a policy framework by midwifery training regulators is required by countries.

## Data Availability

The datasets generated and/or analysed during the current study are not publicly available due to the confidentiality of the data but are available from the corresponding author on request.

## References

[1] Renfrew MJ, McFadden A, Bastos MH, Campbell J, Channon AA, Cheung NF (2014). Midwifery and quality care: findings from a new evidence-informed framework for maternal and newborn care. Lancet.

[2] World Health Organization, United Nations Population Fund, International Confederation of Midwives. The State of the World’s Midwifery 2021: Building a health workforce to meet the needs of women, newborns and adolescents everywhere 2021. https://www.unfpa.org/publications/sowmy-2021.

[3] Filby A, McConville F, Portela A (2016). What prevents quality midwifery care? A systematic mapping of barriers in low and middle income countries from the provider perspective. PLoS ONE.

[4] WHO. Strengthening quality midwifery education for Universal Health Coverage 2030: Framework for action 2019. https://www.who.int/publications/i/item/9789241515849.

[5] United Nations Population Fund, International Confederation of Midwives, World Health Organization. The State of the World’s Midwifery 2021: Building a health workforce to meet the needs of women, newborns and adolescents everywhere 2021. https://www.unfpa.org/publications/sowmy-2021.

[6] International Confederation of Midwives. ICM Global Standards for Midwifery Education. (Revised 2021) 2021. https://www.internationalmidwives.org/assets/files/education-files/2021/10/global-standards-for-midwifery-education_2021_en-1.pdf.

[7] WHO. Midwifery educator core competencies: building capacities of midwifery educators 2014. https://www.who.int/hrh/nursing_midwifery/14116_Midwifery_educator_web.pdf.

[8] Gavine A, MacGillivray S, McConville F, Gandhi M, Renfrew MJ (2019). Pre-service and in-service education and training for maternal and newborn care providers in low-and middle-income countries: an evidence review and gap analysis. Midwifery.

[9] Shikuku DN, Tallam E, Wako I, Mualuko A, Waweru L, Nyaga L (2022). Evaluation of capacity to deliver Emergency Obstetrics and Newborn Care updated midwifery and reproductive health training curricula in Kenya: before and after study. Int J Afr Nurs Sci.

[10] International Confederation of Midwives. Global Standards for Midwifery Regulation. 2011. https://www.internationalmidwives.org/assets/files/regulation-files/2018/04/global-standards-for-midwifery-regulation-eng.pdf. 10.1016/j.midw.2011.04.001 21550149

[11] World Health Organization. Global strategic directions for nursing and midwifery 2021–2025. Geneva: World Health Organization. 2021. https://iris.who.int/bitstream/handle/10665/344562/9789240033863-eng.pdf?sequence=1.

[12] Smith RM, Gray JE, Homer CSE. Common content, delivery modes and outcome measures for faculty development programs in nursing and midwifery: a scoping review. Nurse Educ Pract. 2023:103648. 10.1016/j.nepr.2023.103648 37121027

[13] Nursing and Midwifery Board of Australia. Registration standard: Continuing professional development 2016 3rd January 2022. https://www.nursingmidwiferyboard.gov.au/Registration-Standards/Continuing-professional-development.aspx.

[14] International Confederation of Midwives. Essential competencies for midwifery practice: 2019 Update. 2019.

[15] Baloyi OB, Jarvis MA (2020). Continuing Professional Development status in the World Health Organisation, Afro-region member states. Int J Afr Nurs Sci.

[16] Mack HG, Golnik KC, Murray N, Filipe HP. Models for implementing continuing professional development programs in low-resource countries. MedEdPublish. 2017;6(1). 10.15694/mep.2017.000018 PMC10885220 38406456

[17] Lucas A (2012). Continuous professional development, friend or foe?. Br J Midwifery.

[18] Ingwu JA, Efekalam J, Nwaneri A, Ohaeri B, Israel C, Chikeme P (2019). Perception towards mandatory continuing professional development programme among nurses working at University of Nigeria Teaching Hospital, Enugu-Nigeria. Int J Afr Nurs Sci.

[19] Hasumi T, Jacobsen KH (2014). Healthcare service problems reported in a national survey of South africans. Int J Qual Health Care.

[20] Giri K, Frankel N, Tulenko K, Puckett A, Bailey R, Ross H. Keeping up to date: continuing professional development for health workers in developing countries. IntraHealth Int. 2012.

[21] Botha A, Booi V, editors. mHealth implementation in South Africa. 2016 IST-Africa Week Conference; 2016: IEEE.

[22] World Continuing Education Alliance (WCEA). World Continuing Education Alliance: About us2022 3rd January 2022. https://lmic.wcea.education/about-us/.

[23] West F, Homer C, Dawson A (2016). Building midwifery educator capacity in teaching in low and lower-middle income countries. A review of the literature. Midwifery.

[24] van Wyk JM, Wolvaardt JE, Nyoni CN (2020). Evaluating the outcomes of a faculty capacity development programme on nurse educators in sub-saharan Africa. Afr J Health Professions Educ.

[25] Frantz JM, Bezuidenhout J, Burch VC, Mthembu S, Rowe M, Tan C (2015). The impact of a faculty development programme for health professions educators in sub-saharan Africa: an archival study. BMC Med Educ.

[26] Fullerton JT, Johnson PG, Thompson JB, Vivio D (2011). Quality considerations in midwifery pre-service education: exemplars from Africa. Midwifery.

[27] Shikuku DN, Tallam, E., Wako, I., Mualuko, A., Waweru, L., Nyaga, L., ... & Ameh, C. Evaluation of capacity to deliver Emergency Obstetrics and Newborn Care updated midwifery and reproductive health training curricula in Kenya: Before and after study. 2022

[28] Shikuku DN, Jebet J, Nandikove P, Tallam E, Ogoti E, Nyaga L (2022). Improving midwifery educators’ capacity to teach emergency obstetrics and newborn care in Kenya universities: a pre-post study. BMC Med Educ.

[29] Akiode A, Fetters T, Daroda R, Okeke B, Oji E (2010). An evaluation of a national intervention to improve the postabortion care content of midwifery education in Nigeria. Int J Gynecol Obstet.

[30] Nursing Council of Kenya. Continuing Professional Development guidelines. Nursing Council of Kenya; 2021.

[31] Nursing and Midwifery Council of Nigeria. Promoting & Maintaining Excellence in Nursing Education and Practice: Functions2022. https://www.nmcn.gov.ng/function.html.

[32] Ministry of Health. Kenya Health Policy 2014–2030: Towards attaining the highest standard of health 2014. http://publications.universalhealth2030.org/uploads/kenya_health_policy_2014_to_2030.pdf.

[33] Orsmond GI, Cohn ES (2015). The distinctive features of a feasibility study: objectives and guiding questions. OTJR: Occupation Participation Health.

[34] Bowen DJ, Kreuter M, Spring B, Cofta-Woerpel L, Linnan L, Weiner D (2009). How we design feasibility studies. Am J Prev Med.

[35] Arain M, Campbell MJ, Cooper CL, Lancaster GA (2010). What is a pilot or feasibility study? A review of current practice and editorial policy. BMC Med Res Methodol.

[36] Kirkpatrick DL. Implementing the four levels: A practical guide for effective evaluation of training programs: Easyread super large 24pt edition: ReadHowYouWant. com; 2009.

[37] Warfa A-RM (2016). Mixed-methods design in biology education research: Approach and uses. CBE—Life Sci Educ.

[38] Creswell JW, Creswell JD. Research design: qualitative, quantitative, and mixed methods approaches. Sage; 2017.

[39] Creswell JW, Clark VLP (2018). Designing and conducting mixed methods research.

[40] NCK Online CPD Portal: Continuous Professional Development [Internet]. 2021. https://osp.nckenya.com/cpd?.

[41] Nursing and Midwifery Council of Nigeria. Promoting & maintaining Excellence in nursing education and practice: Renewal of License. 2022. Available from. https://www.nmcn.gov.ng/renewal.html.

[42] Warren N, Gresh A, Mkhonta NR, Kazembe A, Engelbrecht S, Feraud J et al. Pre-service midwifery education in sub-saharan Africa: a scoping review. Nurse Educ Pract. 2023:103678. 10.1016/j.nepr.2023.103678 37413740

[43] Malterud K, Siersma VD, Guassora AD (2016). Sample size in qualitative interview studies: guided by information power. Qual Health Res.

[44] Muellmann S, Brand T, Jürgens D, Gansefort D, Zeeb H (2021). How many key informants are enough? Analysing the validity of the community readiness assessment. BMC Res Notes.

[45] Hennink M, Kaiser BN (2022). Sample sizes for saturation in qualitative research: a systematic review of empirical tests. Soc Sci Med.

[46] Shumway JM, Harden RM (2003). AMEE Guide 25: the assessment of learning outcomes for the competent and reflective physician. Med Teach.

[47] Braun V, Clarke V (2006). Using thematic analysis in psychology. Qualitative Res Psychol.

[48] Erlandsson K, Doraiswamy S, Wallin L, Bogren M (2018). Capacity building of midwifery faculty to implement a 3-years midwifery diploma curriculum in Bangladesh: A process evaluation of a mentorship programme. Nurse Educ Pract.

[49] Erlandsson K, Byrskog U, Osman F, Pedersen C, Hatakka M, Klingberg-Allvin M (2019). Evaluating a model for the capacity building of midwifery educators in Bangladesh through a blended, web-based master’s programme. Global Health Action.

[50] Hatakka M, Osman F, Erlandsson K, Byrskog U, Egal J, Klingberg-Allvin M (2019). Change-makers in midwifery care: exploring the differences between expectations and outcomes—A qualitative study of a midwifery net-based education programme in the Somali region. Midwifery.

[51] Erlandsson K, Osman F, Hatakka M, Egal JA, Byrskog U, Pedersen C (2017). Evaluation of an online master’s programme in Somaliland. A phenomenographic study on the experience of professional and personal development among midwifery faculty. Nurse Educ Pract.

[52] Bogren M, Rosengren J, Erlandsson K, Berg M (2019). Build professional competence and equip with strategies to empower midwifery students–an interview study evaluating a simulation-based learning course for midwifery educators in Bangladesh. Nurse Educ Pract.

[53] Msosa A, Msiska M, Mapulanga P, Mtambo J, Mwalabu G. Simulation-based education in classroom and clinical settings in sub-saharan Africa: a systematic review. Higher Education, Skills and Work-Based Learning; 2023.

[54] Ameh CA, White S, Dickinson F, Mdegela M, Madaj B, van den Broek N (2018). Retention of knowledge and skills after emergency Obstetric Care training: a multi-country longitudinal study. PLoS ONE.

[55] Evans C, Razia R, Cook E (2013). Building nurse education capacity in India: insights from a faculty development programme in Andhra Pradesh. BMC Nurs.

[56] Koto-Shimada K, Yanagisawa S, Boonyanurak P, Fujita N (2016). Building the capacity of nursing professionals in Cambodia: insights from a bridging programme for faculty development. Int J Nurs Pract.

[57] Kitema GF, Laidlaw A, O’Carroll V, Sagahutu JB, Blaikie A. The status and outcomes of interprofessional health education in sub-saharan Africa: a systematic review. J Interprof Care. 2023:1–23. 10.1080/13561820.2023.2168631 36739570

[58] Ladur AN, Kumah EA, Egere U, Mgawadere F, Murray C, Ravit M et al. A blended learning approach for capacity strengthening to improve the quality of integrated HIV, TB, and Malaria services during antenatal and postnatal care in LMICs: a feasibility study. medRxiv. 2023:2023.05. 04.23289508. 10.1186/s12909-024-06633-2 PMC11715178 39780210

[59] World Continuing Education Alliance (WCEA). Improving Health Outcomes: WCEA delivering sustainable solutions for CPD & lifelong learning2023 26th December 2023. https://wcea.education/.

